# Dialysis-Associated Hypertension Treated with Telmisartan – *DiaTel:* A Pilot, Placebo-Controlled, Cross-Over, Randomized Trial

**DOI:** 10.1371/journal.pone.0079322

**Published:** 2013-11-18

**Authors:** Matthias Huber, Till Treutler, Peter Martus, Antje Kurzidim, Reinhold Kreutz, Joachim Beige

**Affiliations:** 1 Department of Infectious Diseases and Nephrology, Kuratorium for Dialysis and Transplantation (KfH) Renal Unit, Leipzig, Germany; 2 Department of Clinical Pharmacology and Toxicology, Charité Medical Centre Berlin, Germany; 3 Department of Clinical Statistics and Biometry, Charité Medical Centre Berlin, Germany; Universidad Peruana de Ciencias Aplicadas (UPC), Peru

## Abstract

Treatment of hypertension in hemodialysis (HD) patients is characterised by lack of evidence for both the blood pressure (BP) target goal and the recommended drug class to use. Telmisartan, an Angiotensin receptor blocker (ARB) that is metabolised in the liver and not excreted via HD extracorporeal circuit might be particularly suitable for HD patients. We designed and conducted a randomised, placebo-controlled, double-blind and cross-over trial for treatment of dialysis–associated hypertension with telmisartan 80 mg once daily or placebo on top of standard antihypertensive treatment excluding other Renin-Angiotensin-System (RAS) blockers. In 29 patients after randomization we analysed BP after a treatment period of 8 weeks, while 13 started with telmisartan and 16 with placebo; after 8 weeks 11 continued with telmisartan and 12 with placebo after cross-over, respectively. Patients exhibited a significant reduction of systolic pre-HD BP from 141.9±21.8 before to 131.3±17.3 mmHg after the first treatment period with telmisartan **or** placebo. However, no average significant influence of telmisartan was observed compared to placebo. The latter may be due to a large inter-individual variability of BP responses reaching from a 40 mmHg decrease under placebo to 40 mmHg increase under telmisartan. Antihypertensive co-medication was changed for clinical reasons in 7 out of 21 patients with no significant difference between telmisartan and placebo groups. Our starting hypothesis, that telmisartan on top of standard therapy lowers systolic office BP in HD patients could not be confirmed. In conclusion, this small trial indicates that testing antihypertensive drug efficacy in HD patients is challenging due to complicated standardization of concomitant medication and other confounding factors, e.g. volume status, salt load and neurohormonal activation, that influence BP control in HD patients.

**Trial Registration:**

Clinicaltrialsregister.eu 2005-005021-60

## Introduction

The prevalence of arterial hypertension in patients with chronic kidney disease stage 5 (CKD 5) receiving haemodialysis (HD) treatment ranges between 60 to 80% [1]. The burden of mortality of CKD 5 patients compared to the general population approaches a 100fold risk increase in epidemiological studies [2], with elevated blood pressure (BP) being only one factor among multiple other factors influencing net cardiovascular risk of that population. BP amplitudes (pulse pressure) and fluctuation between dialysis sessions have been found to have a particular impact on elevated risk [3]. However, no clear threshold values as targets for antihypertensive therapy have been currently defined in HD patients and no guidelines are available for antihypertensive therapy in these patients since BP has not been found to predict outcome in some studies [4]. Recent literature regarding BP variability show an association with atherosclerotic complications [5]. Therapeutic compounds which inhibit the Renin-Angiotensin-System (RAS), e.g. ACE inhibitors (ACE-I) or Angiotensin receptor blockers (ARB) have proven protective cardiovascular effects [6]; [7]. Interestingly, such evidence, while widely recognized in the general hypertensive population and in patients with increased cardiovascular risk, could not be affirmed in CKD 5 patients on maintenance HD treatment, since data are scarce in that field. Only few non-controlled studies addressed surrogates of cardiovascular risk and pharmacokinetics in HD patients so far. The pharmacokinetics including the elimination of the ARB olmesartan, was not influenced by HD in a previous report, but the drug reduced oxidative stress compared to amlodipin [8]–[10]. Telmisartan lowered BP and oxidative stress in HD patients in one open-label, non-controlled study [11], but not yet in a randomised controlled trial. Telmisartan is an attractive candidate for treatment of dialysis-associated hypertension, since it is metabolised and inactivated in the liver and subsequently eliminated predominately via the hepatic route. Moreover, in-vitro experiments demonstrated no excretion of telmisartan by the HD procedure via the dialysis filter [12]. Consequently and given its half-life of 14–18 hours once daily dosing of telmisartan in HD patients seems feasible. On the other hand, no controlled study has been conducted so far targeting BP in HD patients by using telmisartan. This relates probably to the difficulties of clinical management of dialysis-associated hypertension. Albeit the majority of patients are hypertensive, medication mostly cańt be standardized and confounding factors such as volume status, salt load and neurohormonal activation do show fluctuations over time. Our pilot study therefore aimed to explore pre-conditions for a controlled RCT. Thus, we tested an approach using a double-blind, randomized, cross-over controlled design to study the effect of telmisartan compared to placebo on top of a standard antihypertensive medication without treatment of other RAS blockers in HD patients.

## Materials and Methods

### Patient population

The protocol of the trial was approved by the Ethical Committee (EC) of the University Leipzig, Germany (approval dated November 1^st^ 2006) and the responsible federal pharmacovigilance authority (BfArM, dated April 16^th^ 2007). EUDRACT study registration number is 2005-005021-60 and the study is accessible within the European Clinical Trial Database (https://www.clinicaltrialsregister.eu/ctr-search/trial/2005-005021-60/DE). This manuscript was prepared according to the recommendations of the CONSORT statement for clinical trials reporting. Patients were recruited in one HD centre (Leipzig, Saxony, Germany, 150 patients overall) between 2008 and 2010. Patients were eligible for study if they had a clinical indication for antihypertensive drug treatment and were receiving maintenance HD. Detailed inclusion and exclusion criteria are given in Table 1. Before inclusion, all patients gave written informed consent using pre-specified information and consent paper sheets approved by the EC. For regulation of BP and HD dry weight of patients, clinical surrogates like absence of cramps and headache along with maximized ultrafiltration, absence of peripheral oedema, absence of dilated vena cava and absence of (X-ray proven) pulmonary fluid overload were used. Antihypertensive drug treatment according to international guidelines [13] [14] targeting a systolic BP between 150 and 130 mmHg before HD treatment and a systolic BP drop of not more than 20 mmHg after end of HD sessions were applied. Patients with RAS blockers in their standard medication were allowed to be recruited after at least 2 weeks of RAS blocker washout. If BP exceeded BP exclusion criteria during RAS blocker washout, patients were continued on RAS blocker medication but not included in the study. Thus, patients included in the study were either RAS blocker naive or at least 2 weeks free of RAS blocker medication. Antihypertensive medication other than RAS blockers was allowed to change during the study if measured BP values approached the given escape BP criteria or for clinical reasons at the discretion of the treating physicians.

**Table 1:** Inclusion and exclusion criteria.

	Inclusion Criteria	Exclusion Criteria
Age (Years)	18 to 80	
Blood pressure	Any with regard to exclusion criteria	Systolic BP <120 mmHg without antihypertensive medication, Systolic BP >170 mmHg with antihypertensive medication
Antihypertensive medication	Standard antihypertensive treatment after wash-out of RAS blockers	Clinical indications for RAS blockers other than hypertension (congestive heart failure, myocardial infarction)
	Written informed consent	Limited legal capacity
Co morbidity and individual requirements		Clinical apparent infectious diseases
		Clinical apparent malignant diseases with life expectancy <12 months
		Incompatibility of study medication
		Pregnancy, non-ability or no willingness of birth control in female patients of child-bearing potential

### Study design, intervention and variables

**Figure 1:**
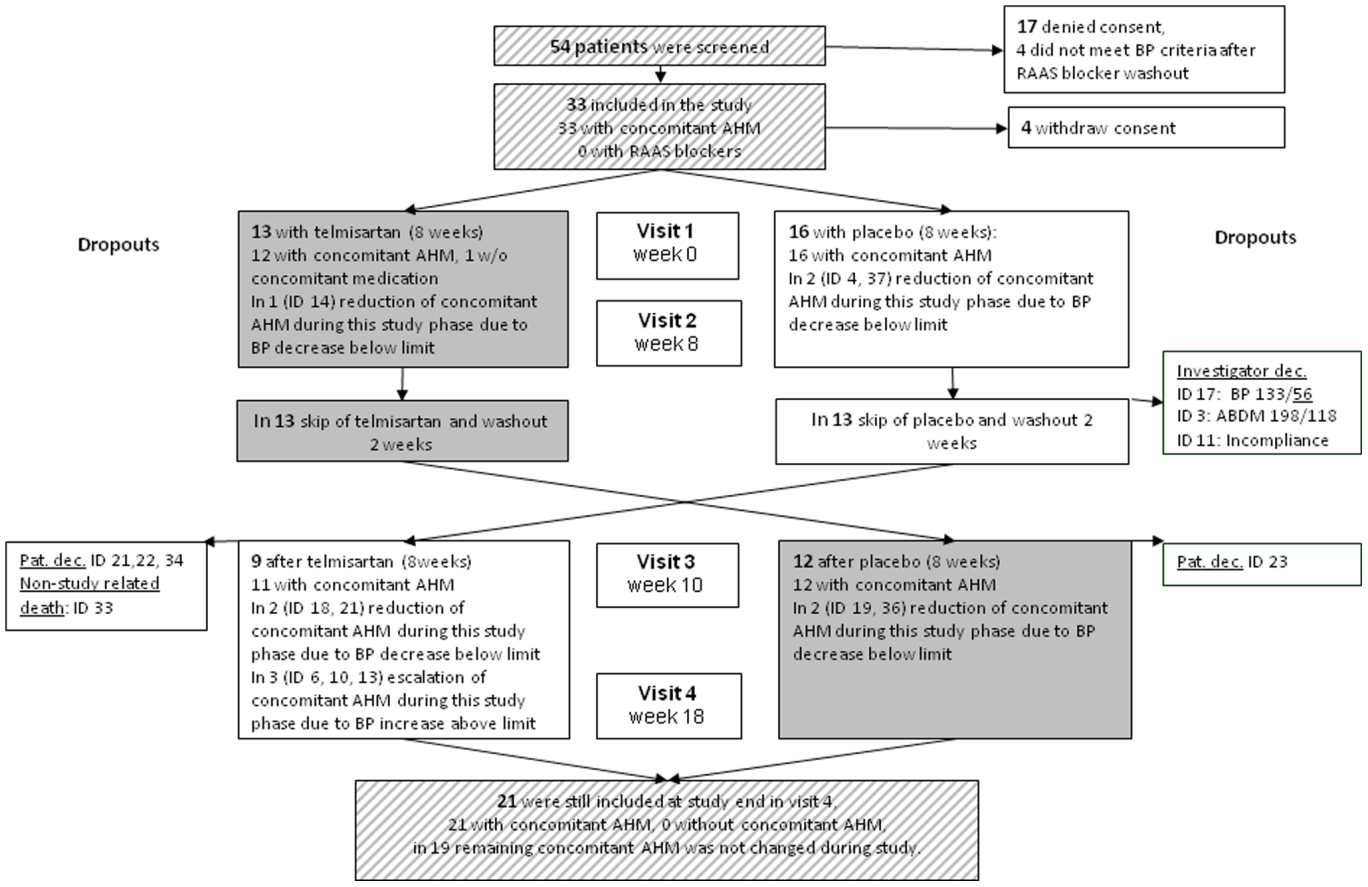
Study flow chart. Study flow chart with individual causes of study interruptions and dropouts.

The study was placebo-controlled, double-blinded and randomized using a cross-over design. After screening, wash-out of RAS blockers (if appropriate) and informed consent, patients were allocated to either first placebo or telmisartan treatment. Envelopes containing results of pre-defined randomization lists were used for randomisation. For study medication, tablets of either 80 mg telmisartan (Kinzalmono®, Bayer Healthcare, Leverkusen, Germany) or matching placebo (P-Tabletten®, Winthrop Pharma, Fürstenfeldbruck, Germany) were used. Both physicians and patients were blinded for treatment. After 8 weeks of treatment (telmisartan or placebo) and a subsequent period of 2 weeks of wash-out, patients were crossed over to the alternative treatment period (placebo or telmisartan). BP measurements pre and after HD were conducted at the beginning and end of either treatment period, i.e. every patient had four blood pressure visits; before and after of each telmisartan and placebo period. The study flow chart is given in Fig. 1. BP values exceeding or undershooting inclusion levels (Table 1) at 2 subsequent occasions during the study were considered as escape criteria. Thus, a recording of BP above or below thresholds in two subsequent measurements resulted in drop-out. Adjustment for BP effect of other-than-study medication was done by: (i) computation of an “adjusted BP” (Table 2) by adding 10 mmHg for full-dosed and 5 mmHg for not full-dosed medication to the measured systolic BP according to Tobin et al. [15], (ii) counting and displaying the number of non-study BP drugs by 1 point for any full dosed and 0.5 points for any not full dosed medication (Table 2).

**Table 2:** Anthropometrical and epidemiological characterization of included patients.

	Start Telmisartan N = 13	Start Placebo N = 16
Age (Years)	59.3±18.0	56.9±13.1
Male gender [n, (%)]	10 (77)	13 (81)
History of hypertension (Years)	18±11.4	15.9±9.6
Time on HD at screening – Vintage (Years)	4.31±3.70	2.56±2.31
Dry weight (kg)	78.3±20.1	78.2±16.6
Ultrafiltration per session (mL)^1^	1900±401	2700±621
Residual diuresis (mL)	1000±606	717±601
Diabetics [n, (%)]	5 (38.1)	5 (31.3)
Left ventricular hypertrophy [n, (%)]	2 (22.2)	4 (25)

***in telmisartan periods, p = 0.058.***

### Study end-points

The office systolic BP before HD sessions was the primary study end-point. BP after HD session and ambulatory blood pressure (ABDM) results were regarded as secondary end-points. Office BP was measured by an automated upper arm BP device (Boso™, Germany) or an incorporated BP module of dialysis machines by nursing staff before HD initiation. ABDM was measured by recorders and software of Custo Diagnostics™, Germany. All BP devices were subjected to regular calibration and maintenance of authorities and manufacturers.

Sample size estimation was based on a conservative expectation (data from Shimada et al. [11]) of a telmisartan effect of 8 mmHg BP reduction with an intra-individual standard deviation of 15 mmHg. On that effect size, 30 patients were needed to confirm superiority of telmisartan vs. placebo with 5% probability of error.

### Adverse events and safety measurements

Adverse events (AE) and severe adverse events (SAE) were classified according standard regulations of good clinical praxis (GCP). Any hospitalisation or death during study was regarded as SAE and relationship to study medication was attributed as “yes” or “no”. Unexpected, i.e. clinical significant change of laboratory parameters or any clinical signs of patients were regarded as AE. For monitoring of safety, standard routine procedures of maintenance dialysis were used. They consisted of monthly control of blood count, liver enzymes and electrolytes. Every 3 months parameters of bone metabolism were investigated.

### Statistical analysis

Systolic BP reflecting the primary endpoint was measured at visits 1, 2, 3, and 4. In a first step, the differences between visit 2 and visit 1 (D21) and between 4 and visit 3 (D43) were calculated. The variables D21 and D43 entered a *Manova* model with time as within subject factor (df  = 1), sequence of treatment (telmisartan first vs. placebo first) as between subject factor (df  = 1) and time treatment interaction (df  = 1). In this model, the interaction term represents the difference between both treatments whereas the between subject factor represents a possible time treatment interaction. The primary analysis population was the intention to treat population. Per protocol data are given additionally and with particular identification (Table 3). Missing values of visits 2 and 4 were replaced by measured values at visits 1 and 3, respectively “with last observation carried over (LOF)” within treatment phases only. The level of significance was 0.05 (two-sided) for each statistical test. Analyses were calculated using commercially available software (SPSS for Windows, Release 18.0).

**Table 3:** BP end-points, dialysis dry weight and interdialytic weight gain before and after treatment periods (per-protocol results highlighted).

	Before telmisartan 80mg once daily	After telmisartan 80mg once daily	Before placebo	After placebo
BP sys pre dialysis (mmHg)	131.0±17.3	132.5±20.3	142.0±19.7	135.7±19.5
BP sys pre dialysis, adjusted for medication (mmHg)	147.4±20.9	150.7±28.5	160.5±24.1	155.3±25.7
BP dia pre dialysis (mmHg)	72.8±10.7	74.4±16.0	78.4±13.8	76.0±14.1
Non-study antihypert. med. (n)	2.08±1.38	2.17±1.40	2.79±1.12	2.77±1.74
HR pre dialysis (1/min)	66.2±10.7	62.7±10.1	65.5±10.8	72.6±10.6
BP sys after dialysis (mmHg)	124.8±26.8	129.2±25.4	135.4±20.4	127.6±21.2
BP dia after dialysis (mmHg)	67.4±13.9	73.9±14.2	72.6±14.3	71.8±10.9
ABDM sys day (mmHg)	132.4±8.8	133.4±16.6	139.8±19.3	136.3±18.5
ABDM dia day (mmHg)	69.6±14.1	75.2±15.2	78.6±11.0	75.5±16.2
ABDM sys night (mmHg)	128.6±16.4	128.4±17.4	133.2±25.6	131.0±28.6
ABDM dia night (mmHg)	69.6±14.1	75.2±15.2	78.6±11.0	75.5±16.2
HD dry weight (kg; range)	77.4±19.8; 57…136	77.1±20.3; 54.5…135	76.4±14.7; 56…121	76.9±15.2; 56…123
BP sys pre dialysis (mmHg)^1^	131.8±13.6	134.3±18.5	137.5±15.3	133.6±20.1
BP sys pre dialysis, adjusted for medication (mmHg)^1^	150.1±18.1	154.3±25.9	157.5±21.4	155.2±25.7
BP dia pre dialysis (mmHg) 1	73.4±10.0	75.7±13.5	78.0±14.0	71.4±11.6
Dose increase of non-study antihypertensive drugs (n)^1^		3		0
Dose decrease of non-study antihypertensive drugs in (n)^1^		3		4
Mean dry weight change per visit (kg)^1^	0.481±0.375		0.479±0.377	

1 Per protocol, all other intention to treat.

## Results

### Population

Anthropometrical and clinical characterization of patients of the intention-to-treat population is given in Table 2. There were no significant differences between patients starting with either telmisartan or placebo treatment. The same was true for the per-protocol population (data not shown).

### End-points

**Figure 2:**
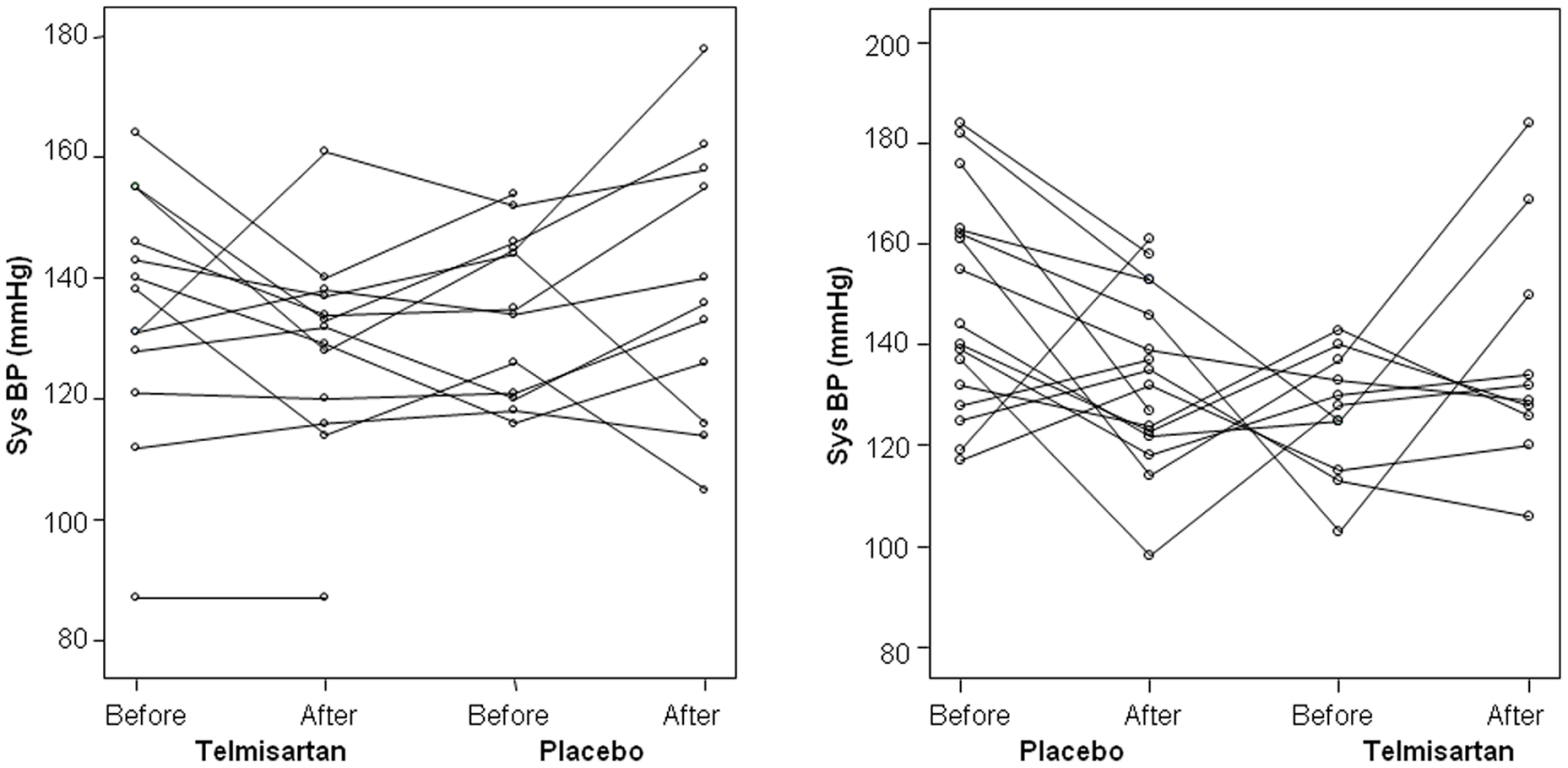
Individual courses of blood pressure. Courses of systolic BP before HD in patients starting with 80(left panel) or placebo (right panel). Lines are not completely drawn through if patients dropped out during study.

Course of systolic office BP pre HD in either placebo or telmisartan starting group is given in Fig. 2a and 2b, respectively.

After telmisartan treatment, in eleven patients systolic office BP decreased compared to 10 patients in whom systolic office BP increased. After placebo treatment, systolic office BP decreased in 14 and increased in 11 patients. Means of office BP and ABDM values showed no significant differences when compared between study phases (Table 3). This was also true, if LOCF data or per-protocol data of the primary end-point were analysed.

However, when analysing BP values with regard to time and not to treatment, there was a significant study time effect on BP. Between visits 1 and 2 in both groups, systolic office BP fell from 141.9±21.8 to 131.3±17.3 and increased from 130.6±13.4 to 138.1±22.5 between visits 3 and 4 (all units mmHg; p = 0.006). Although there were was a trend towards a greater BP decrease in response to placebo (mean −6.4 mmHg), this was not statistically significant. Concomitant non-study antihypertensive medication was changed in 7 patients, but this was not significantly different between telmisartan or placebo treatment. Overall, with regard to both the primary (systolic office BP) and secondary (after HD office and ABDM) BP end-points, this pilot study could not confirm the hypothesis, that treatment with telmisartan results in a significant decrease of BP in patients treated with haemodialysis.

### Safety and adverse events

Overall four SAE were registered during the study. One occurred before start of treatment, two during telmisartan and one during placebo treatment. One SAE during telmisartan resulted in a fatal outcome but was not classified as study-related. The female patient died from pulmonary embolism. Other SAE were also considered not to be study-related. The number of AE was 58, including 4 before either phase, 24 during telmisartan and 30 during placebo. Leukocyte count under telmisartan were lower compared to placebo at visit 2 (6.42±1.82 vs. 8.17±1.74 giga-particles per liter [GPT/L] p = 0.015), while this was not repeated at visit 4. No single leukocyte count fell below 3.8 GPT/L.

## Discussion

In this randomized, double-blinded, cross-over placebo-controlled pilot study we demonstrated that treatment with 80 mg telmisartan once daily on top of standard non-RAS therapy was not effective to lower systolic office BP and other (secondary) BP measures in HD patients. This negative result was attributed to remarkable individual BP variations in the overall group of HD patients during the course of the study and thus affected both telmisartan and placebo treated patients. Such individual changes exceeded several times the mean population changes observed in the entire cohort and reached BP decreases of up to 40 mmHg during placebo and increases up to 40 mmHg during the treatment period with telmisartan. In this regard it should not be dismissed that the non-study antihypertensive medication had to be changed in 7 patients. The proportion of changed co-medications, however, was not different between telmisartan and placebo treatment groups. The study was characterized by a remarkable and significant study time effect on BP which might constitute an additional confounder in the frame of the chosen time-dependent cross-over design.

In 6 of our patients with available plasma concentrations after taking telmisartan, levels did not exceed 200 ng/mL, which was substantially lower compared to data of *Stangier* et al. [12], who administered 120 mg to their patients. Therefore, our dosage of 80 mg might be too low to exert a substantial blood pressure lowering effect.

Our overall results do not indicate that the null-hypothesis was confirmed and that telmisartan therefore could not be considered as an effective BP lowering drug in HD patients with certain statistical significance. Our pilot study rather suggests that inclusion of a relatively small number of patients in one HD centre in is not powerful enough to yield reliable results. This is important because we did not note any safety issues related to the use of telmisartan (i.e. hyperkalemia). At variance with the current data, in an uncontrolled open-label study telmisartan was effective in 14 patients to lower BP, brain natriuretic peptide and oxidative stress [11]. Such different outcomes illustrate the obstacles related to design and conduction of RCTs in HD patients presenting with a wide repertoire of clinical confounders affecting BP control. Along with *Shimada* et al. we do also acknowledge the need of a well-designed, appropriate powered RCT to test the efficacy of telmisartan and other ARB in HD patients. Nevertheless, the present study reveals some important insights by demonstrating the impact of BP variations and the role of concomitant (non-study) medication in this setting of HD patients. Consequently, we would choose a different design for subsequent RCT addressing the issue of BP control in HD patients. Antihypertensive drugs should be fixed at pre-study dosage during study and change of medication should only be allowed if BP changes in a clinical significant matter. This approach is however challenging, since patients and physicians use to stay within systolic BP thresholds in the range between 120 and 140 mmHg, although they are presently not confirmed to be relevant for mortality outcome in dialysis populations. Thus, intensive further research should not only address appropriate medication but the question of an optimal BP (and allowed study BP target range) per se in HD patients.

## Supporting Information

Checklist S1 CONSORT Checklist.
(DOC)

Protocol S1 Trial Protocol.
(PDF)
