# Supplementary material for: Dialysis-Associated Hypertension Treated with Telmisartan – DiaTel: A Pilot, Placebo-Controlled, Cross-Over, Randomized Trial
Source: PLoS One. 2013 Nov 18;8(11):e79322. doi: 10.1371/journal.pone.0079322 (PMC3832496; doi:10.1371/journal.pone.0079322)
Supplement: Protocol S1 — Trial Protocol. (PDF) [file pone.0079322.s002.pdf]

REQUEST FOR AUTHORISATION OF A CLINICAL TRIAL ON A MEDICINAL PRODUCT FOR HUMAN USE TO THE COMPETENT AUTHORITIES AND FOR OPINION OF THE ETHICS COMMITTEES IN THE COMMUNITY

For official use:

Date of receiving the request :<br><br>Date of request for information to make it valid :	Date of request for additional information :	Grounds for non acceptance/ negative opinion :❏<br><br>Give date :
Date of valid application :<br><br>Date of start of procedure :	Date of receipt of additional / amended information :	Authorisation/ positive opinion :❏<br><br>Give date:
Competent authority registration number :<br><br>Ethics Committee registration number :		Withdrawal of application : ❏<br><br>Give date :

To be filled in by the applicant:

The questions in this form for the request for authorisation from the Competent Authority are also relevant for the opinion from an Ethics Committee (it represents module 1 of the form for applying to an ethics committee) and can be used as part of that application. Please indicate the relevant purpose in a box below.

REQUEST FOR AUTHORISATION TO THE COMPETENT AUTHORITY: ✓

A. TRIAL IDENTIFICATION

A.1 Member State in which the submission is being made : GERMANY

A.2 EudraCT number[1]: **2005-005021-60**

A.3 Full title of the trial:
Monozentrische, randomisierte, einfach verblindete, Placebo kontrollierte Studie im Cross over Design zur Untersuchung der Wirkung von Telmisartan (Kinzalmono 80 mg) auf Blutdruck und Endothelfunktion bei Patienten unter Hämodialysebehandlung.

A.4 Sponsor’s protocol code number[2]: **01**
Sponsor’s protocol version[2]: **02**
Sponsor’s protocol date[2]: **2005-10-18**

A.5 Name or abbreviated title of the trial where available:
DIATEL Studie

A.6 ISRCTN number[3], if available :
A.7 Is this a resubmission ? yes ❏ no ✓
If Yes, indicate the resubmission letter[4] : **First submission**

---

[1] Append the EudraCT number confirmation receipt

[2] Any translation of the protocol should be assigned the same date and version as those in the original document.

[3] International Standard Randomised Controlled Trial Number. Sponsors may wish to use an International Standard Randomised Controlled Trial Number (ISRCTN) to identify their trial in addition to the EudraCT number; for instance if their trial is part of a multinational trial with sites outside the Community. They can obtain the number and guidance from the Current Controlled Trials website http://www.controlled-trials.com/isrctn to which there is a link from the

EudraCT database website http://www.eudract.emea.eu.int. When available they should provide it in Section A.6 of the application form.

[4] For a resubmission following previous withdrawal of an application or unfavourable opinion of an ethics committee, or previous withdrawal of an application or refusal of a request by the competent authority, enter a letter in the sequence, A for first resubmission, B for second, C for third et seq.

B. IDENTIFICATION OF THE SPONSOR RESPONSIBLE FOR THE REQUEST

B.1 Sponsor

B.1.1 Name of organisation :	Prof. Dr. R. Kreutz Leiter der Abteilung Klinische Pharmakologie der Charite Berlin
B.1.2 Name of the person to contact:	Matthias Huber
B.1.3 Address :	Hindenburgdamm 30<br>Berlin<br>12200<br>GERMANY
B.1.4 Telephone number :	
B.1.5 Fax number :	
B.1.6 e-mail:	

B.3 Status of the sponsor :	B.3.1 commercial[6] ❑	B.3.2 non commercial ✓

B.2 Legal representative[5] of the sponsor in the Community for the purpose of this trial (if different from the sponsor)

B.2.1 Name of organisation :	
B.2.2 Name of the person to contact:	
B.2.3 Address :	
B.2.4 Telephone number :	
B.2.5 Fax number :	
B.2.6 e-mail:	

[5] : In accordance with article 19 of Directive 2001/20/EC

[6] : A commercial sponsor is a person or organisation that takes responsibility for a trial which at the time of the application is part of the development programme for a marketing authorisation of a medicinal product.

C. APPLICANT IDENTIFICATION, (please tick the appropriate box)

C.1 Request for the competent authority ✓

C.1.1 - Sponsor	✓
C.1.2 - Legal representative of the sponsor	❑
C.1.3 - Person or organisation authorised by the sponsor to make the application.	❑

C.1.4 Complete the details of the applicant below even if they are provided elsewhere on the form:

C.1.4.1 Organisation :	Prof Dr. R. Kreutz Leiter der Abteilung Klinische Pharmakologie der Charite Berlin
C.1.4.2 Name of contact person :	Matthias Huber
C.1.4.3 Address :	Hindenburgdamm 30<br>Berlin<br>12200<br>GERMANY
C.1.4.4 Telephone number :	
C.1.4.5 Fax number :	
C.1.4.6 e-mail:	matthias.huber@charite.de

C.1.5 Request to receive an .xml copy of CTA data :

C.1.5.1 Do you want an .xml file copy of the CTA form data saved on EudraCT ? yes ✓ no ❑

C.1.5.1.1 If Yes provide the e-mail address(es) to which it should be sent (up to 5 addresses) :

matthias.huber@charite.de

C.1.5.1.2 Do you want to receive this via password protected link(s)[7] ? yes ✓ no ❑

If you answer No to question C.1.5.1.2 the .xml file will be transmitted by less secure e-mail link(s)

[7] This requires a EudraLink account. (See www.eudract.emea.eu.int) for details)

D. INFORMATION ON EACH IMP

Information on each 'bulk product' before trial-specific operations (blinding, trial specific packaging and labelling) should be provided in this section for each investigational medicinal product (IMP) being tested including each comparator and each placebo, if applicable. If the trial is performed with several products use extra pages and give each product a sequential number in D.1.1. If the product is a combination product information should be given for each active substance.

D.1 IMP IDENTIFICATION

Indicate which of the following is described below, then repeat as necessary for each of the numbered IMPs to be used in the trial(assign numbers from 1-n):

**D.1.1 This refers to the IMP number :**	PR1
**D.1.2 IMP being tested**	✓
**D.1.3 IMP used as a comparator**	❑

for placebo go directly to D.7

D.2 STATUS OF THE IMP. If the IMP has a marketing authorisation in the Member State concerned by this application but the trade name and marketing authorisation holder are not fixed in the protocol, go to section D.2.2.

D.2.1 Has the IMP to be used in the trial a marketing authorisation ?	yes ✓ no ❑
D.2.1.1 If yes to D.2.1, specify for the product to be used in the trial :	
D.2.1.1.1 Trade name[9]	Kinzalmono 80 mg Tabletten
D.2.1.1.2 Name of the MA holder[9]	Bayer AG
D.2.1.1.3 MA number (if MA granted by a Member State)[9]	EU/1/98/091/005
D.2.1.1.4 Is the IMP modified in relation to its MA ?	yes ✓ no ❑
D.2.1.1.4.1 If Yes, please specify	The whole tablet is carefully inserted in a capsule. The capsule is identical to that used for the preparation of placebo. The major ingredient of the capsule is gelatine.
D.2.1.2 Which country granted the MA ?	GERMANY
D.2.1.2.1 Is this the Member State concerned with this application ?	yes ✓ no ❑
D.2.1.2.2 Is this another Member State ?	yes ❑ no ✓

**D.2.2 Situations where an IMP to be used in the CT has a MA in the MS concerned , but the protocol allows that any brand of the IMP with a MA in that MS be administered to the trial subjects and it is not possible to clearly identify the IMP(s) in advance of the trial start**	
D.2.2.1 In the protocol, is treatment defined only by active substance ?<br><br>D.2.2.1.1 If Yes, give active substance in D.3.8 or D.3.9	yes ❑ no ❑
D.2.2.2 In the protocol, do treatment regimens allow different combinations of marketed products used according to local clinical practice at some or all investigator sites in the MS ?<br><br>D.2.2.2.1 If Yes, give active substance in D.3.8 or D.3.9	yes ❑ no ❑
D.2.2.3 The products to be administered as IMPs are defined as belonging to an ATC group[9].<br><br>D.2.2.3.1 If Yes, give the ATC group of the applicable authorised codes in the ATC code field (level 3 or the level that can be defined) in D.3.3	yes ❑ no ❑
D.2.2.4 Other :	yes ❑ no ❑
D.2.2.4.1 If Yes, please specify :	

**D.2.3 IMPD submitted :**	
D.2.3.1 Full IMPD	yes ❑ no ❑
D.2.3.2 Simplified IMPD[10].	yes ❑ no ❑
D.2.3.3 Summary of product characteristics (SmPC) only	yes ❑ no ❑

**D.2.4 Has the use of the IMP been previously authorised in a clinical trial conducted by the sponsor in the Community ?**	yes ❑ no ✓
D.2.4.1 If Yes, specify which Member States :	

**D.2.5 Has the IMP been designated in this indication as an orphan drug in the Community ?**	yes ❑ no ✓
D.2.5.1 If yes, give the orphan drug designation number[11]**:**	

D.2.6 Has the IMP been the subject of scientific advice related to this clinical trial ? yes ❑ no ✓

D.2.6.1 If Yes to D.2.6 please indicate source of advice and provide a copy in the CTA request :

D.2.6.1.1 From the CHMP [12]? yes ❑ no ❑

D.2.6.1.2 From a MS competent authority ? yes ❑ no ❑

[9] Available from the Summary of Product Characteristics

[10] Provide justification for using simplified dossier in the covering letter.

[11] According to the Community register on orphan medicinal products (Regulation (EC) n° 141/2000) : http://pharmacos.eudra.org/F2/register/orphreg.htm

[12] Committee for Medicinal Products for Human Use of the European Medicines Agency.

D.3 DESCRIPTION OF THE IMP

**D.3.1 Product name where applicable[13] :**	Telmisartan 80 mg
**D.3.2 Product code where applicable[14] :**	
**D.3.3 ATC code, if officially registered[15] :**	C09CA07
**D.3.4 Pharmaceutical form (use standard terms) :**	Capsule

D.3.5 Maximum duration of treatment of a subject according to the protocol :

2 months

D.3.6 Maximum dose allowed (specify : per day or total dose; units and route of administration) :

80 mg

Per day or total dose :	Per day
Units :	80 mg milligram(s)
Route of administration :	Gastroenteral Use
**D.3.7 Route of administration (use standard terms):**	Gastroenteral Use

---

**D.3.8 Name of each active substance (INN or proposed INN if available) :**	Telmisartan
**D.3.9 Other available name for each active substance (CAS [16] , current sponsor code(s), other descriptive name, etc : provide all available) :**	
- CAS	144701-48-4
- Current sponsor code(s)	BIBR 277 SE
- Other Descriptive name	
**D.3.10 Strength (specify all strengths to be used) :**	
D.3.10.1 - concentration unit :	mg milligram(s)
D.3.10.2 - concentration type (“exact number”, “range”, “more than” or “up to”).	equal
D.3.10.3 - concentration number :	80

[13] To be provided only where there is no tradename. This is the name routinely used by the sponsor to identify the IMP in the CT documentation (protocol, IB...)

[14] To be provided only where there is no tradename. This is the code designated by the sponsor which represents the name routinely used by the sponsor to identify the product in the CT documentation. For example, a code may be used for combinations of drugs or drugs and devices..

[15] Available from the Summary of Product Characteristics

[16] Chemical Abstracts Service.

**D.3.11 Type of IMP**	
**Does the IMP contain an active substance :**	
D.3.11.1 - of chemical origin ?	yes ✓ no ❑
D.3.11.2 - of biological / biotechnological origin?[17]	yes ❑ no ✓
**Is this a:**	
D.3.11.3 - Cell therapy medicinal product?[17]	yes ❑ no ✓
D.3.11.4 - Gene therapy medicinal product?[17]	yes ❑ no ✓
D.3.11.5 - Radiopharmaceutical medicinal product ?	yes ❑ no ✓
D.3.11.6 - Immunological medicinal product (such as vaccine, allergen, immune serum)?	yes ❑ no ✓
D.3.11.7 - Plasma derived medical product?	yes ❑ no ✓
D.3.11.8 - Other extractive medical product?	yes ❑ no ✓
D.3.11.9 - Herbal medicinal product?	yes ❑ no ✓
D.3.11.10 - Homeopathic medicinal product?	yes ❑ no ✓
D.3.11.11 - Medicinal product containing genetically modified organisms?	yes ❑ no ✓
• If yes to D.3.11.11	
▪ D.3.11.11.1 Has the authorisation for contained use or release been granted?	yes ❑ no ❑
▪ D.3.11.11.2 Is it pending ?	yes ❑ no ❑
D.3.11.12 - Another type of medicinal product?	yes ❑ no ✓
• D.3.11.12.1 If yes, specify :	

[17] Complete also sections D.4, and where applicable sections D.5 and D.6

D.4. BIOLOGICAL / BIOTECHNOLOGICAL INVESTIGATIONAL MEDICINAL PRODUCTS INCLUDING VACCINES

**D.4.1 Type of product**	
D.4.1.1 - Extractive	yes ❑ no ❑
D.4.1.2 - Recombinant	yes ❑ no ❑
D.4.1.3 - Vaccine	yes ❑ no ❑
D.4.1.4 - GMO	yes ❑ no ❑
D.4.1.5 - Plasma derived products	yes ❑ no ❑
D.4.1.6 - Others	yes ❑ no ❑
D.4.1.6.1 If others, specify :	

D.5 SOMATIC CELL THERAPY INVESTIGATIONAL MEDICINAL PRODUCT (NO GENETIC MODIFICATION)

**D.5.1 Origin of cells**	
D.5.1.1 - Autologous	yes ❑ no ❑
D.5.1.2 - Allogeneic	yes ❑ no ❑
D.5.1.3 - Xenogeneic	yes ❑ no ❑
D.5.1.3.1 - If yes, specify species of origin :	

D.5.2 Type of cells

D.5.2.1 - Stem cells yes ❑ no ❑

D.5.2.2 - Differentiated cells yes ❑ no ❑

D.5.2.2.1 If yes, specify the type (e.g. keratinocytes, fibroblasts, chondrocytes,…) :

D.5.2.3 - Others : yes ❑ no ❑

D.5.2.3.1 If others, specify :

D.6. GENE THERAPY INVESTIGATIONAL MEDICINAL PRODUCTS

D.6.1 Gene(s) of interest :

D.6.2 In vivo gene therapy: ❑

D.6.3 Ex vivo gene therapy : ❑

D.6.4 Type of gene transfer product

D.6.4.1 - Nucleic acid (e.g. plasmid) : yes ❑ no ❑

If yes, specify

D.6.4.1.1 - Naked : yes ❑ no ❑

D.6.4.1.2 - Complexed : yes ❑ no ❑

D.6.4.2 - Viral vector : yes ❑ no ❑

D.6.4.2.1 If yes, specify the type : adenovirus, retrovirus, AAV, …:

D.6.4.3 - Others : yes ❑ no ❑

D.6.4.3.1 If others, specify :

**D.6.5 Genetically modified cells :**	yes ❑ no ❑

If yes, specify origin of the cells :

D.6.5.1 - Autologous :	yes ❑ no ❑
D.6.5.2 - Allogeneic :	yes ❑ no ❑
D.6.5.3 - Xenogeneic :	yes ❑ no ❑

D.6.5.3.1 - If yes, specify species of origin :

D.6.5.4 - Other type of cells (hematopoietic stem cells, …) :	yes ❑ no ❑

-If Yes specify :

D.6.6 Comments on novel aspects of gene therapy investigational product if any (free text) :

D.7 INFORMATION ON PLACEBO (if relevant repeat as necessary)

D.7.1 Is there a placebo: yes ✓ no ❑

D.7.2 This refers to Placebo number (......)	PL1
D.7.3 Pharmaceutical form :	Capsule
D.7.4 Route of administration :	Oral Use
D.7.5 Which IMP is it a placebo for? Specify IMP Number(s) from D.1.1	PR1
D.7.5.1 Composition, apart from the active substance(s) :	
D.7.5.2 - is it otherwise identical to the IMP?	yes ❑ no ✓

D.7.5.2.1- if not, specify major ingredients :

the capsule of placebo is identical to the capsule of the IMP, the major ingredients of placebo which are encapsulated are Lactose and Cellulose

D.8 SITE WHERE THE QUALIFIED PERSON CERTIFIES BATCH RELEASE[18]

This section is dedicated to ***finished*** *IMPs i.e. medicinal products randomised, packaged, labelled and certified for use in the clinical trial. If there is more than one site or more than one IMP is certified, use extra pages and give each IMP its number from Section D.1.1 or D.7.2. In the case of multiple sites indicate the product certified by each site.*

D.8.1 Do not fill in section 8.2 for an IMP that:

- Has an MA in the EU **and**
- Is sourced from the EU market **and**
- Is used in the trial without modification (eg not overencapsulated) **and**
- The packaging and labelling is carried out for local use only as per article 9.2 of the Directive 2005/28/EC (GCP Directive)

If all these conditions are met tick and list the number(s) of each IMP including placebo from sections D.1.1 and D.7.2. to which this applies

---

[18]In accordance with paragraph 38 of Annex 13 of Volume 4 of the Rules Governing Medical Products in the European Union

D.8.2 Who is responsible in the Community for the certification of the finished IMP ? :

**This site is responsible for certification of (list the number(s) of each IMP including placebo concerned from sections D.1.1 and D.7.2) :**	PR1<br>PL1

Please tick the appropriate box:

D.8.2.1 - Manufacturer	✓
D.8.2.2 - Importer	❑

D.8.2.3 Name of the organisation :	TU Dresden, Pharmacy
D.8.2.3.1 Address :	Dresden<br>GERMANY

D.8.2.4 - Give the manufacturing authorisation number :

D.8.2.4.1 If no authorisation, give the reasons :

Where the product does not have a MA in the EU but is supplied in bulk and final packaging and labelling for local use is carried out in accordance with article 9.2 of Directive 2005/28/EC/(GCP Directive) then enter the site where the product was finally certified for release by the Qualified Person for use in the clinical trial at D.8.2 above

E. GENERAL INFORMATION ON THE TRIAL

The section should be used to provide information about the aims, scope and design of the trial. When the protocol includes a sub-study in the MS concerned section E.2.3 should be completed providing information about the sub-study. To identify it check the sub-study in the 'Objective of the trial' question below.

E.1 Medical condition or disease under investigation

E.1.1 Specify the medical condition(s) to be investigated[19] (free text) :

Treatment of hypertension and endothelial dysfunction with Telmisartan in patients with haemodialysis due to chronic renal failure.

E.1.2 MedDRA version, level, term and classification code [20] (repeat as necessary) :

Version	Level	Code	Term

E.1.3 Is any of the conditions to be studied a rare disease[21] ? yes ❑ no ✓

E.2 Objective of the trial

E.2.1 Main objective :

Efficacy and safety of Telmisartan in patients which are haemodialysed due to chronic renal failure

E.2.2 Secondary objectives :

Pharmacokinetics of Telmisartan

E.2.3 Is there a sub-study ? yes ❑ no ✓

E.2.3.1 If Yes, give the full title, date and version of each sub-study and their related objectives :

[19] In the case of healthy volunteer trial, the intended indication for the product under development should be provided.

[20] Applicants are encouraged to provide the MedDRA lower level term if applicable and classification code. These can be accessed from the EMEA EudraCT website (http://www.emea.eu.int)

[21] Points to consider on the calculation and reporting of the prevalence of a condition for Orphan drug designation : COM/436/01 (www.emea.eu.int/htms/human/comp/orphaapp.htm)

**E.3 Principal inclusion criteria** (list the most important)
patients (male or female) with haemodialysis due to chronic renal failure age over 18 years and below 80 years which suffer from hypertension.

**E.4 Principal exclusion criteria** (list the most important)
patients without antihypertensive treatment and blood pressure below 120 mm Hg systolic<br>patients with standardized antihypertensive treatment and blood pressure over 170 mm Hg

**E.5 Primary end point(s) :**
efficacy and safety of Telmisartan in patients with haemodialysis

E.6 Scope of the trial – Tick all boxes where applicable

E.6.1 - Diagnosis	❏
E.6.2 - Prophylaxis	❏
E.6.3 - Therapy	❏
E.6.4 - Safety	✓
E.6.5 - Efficacy	✓
E.6.6 - Pharmacokinetic	✓
E.6.7 - Pharmacodynamic	❏
E.6.8 - Bioequivalence	❏

E.6.9 - Dose Response ❑

E.6.10 - Pharmacogenetic ❑

E.6.11 - Pharmacogenomic ❑

E.6.12 - Pharmacoeconomic ❑

E.6.13 - Others ❑

E.6.13.1 If others, specify :

E.7 Trial type[22] and phase

E.7.1 Human pharmacology (Phase I) yes ❑ no ✓

Is it:

E.7.1.1 First administration to humans yes ❑ no ✓

E.7.1.2 Bioequivalence study yes ❑ no ✓

E.7.1.3 Other yes ❑ no ✓

E.7.1.3.1 If Other, please specify :

E.7.2 Therapeutic exploratory (Phase II) yes ❑ no ✓

E.7.3 Therapeutic confirmatory (Phase III) yes ✓ no ❑

E.7.4 Therapeutic use (Phase IV) yes ❑ no ✓

E.8 Design of the trial

E.8.1 Controlled : yes ✓ no ❑

- If yes, specifiy :

E.8.1.2 Open : yes ❑ no ✓

E.8.1.1 Randomised : yes ✓ no ❑

E.8.1.3 Single blind : yes ✓ no ❑ E.8.1.4 Double blind : yes ❑ no ✓

E.8.1.5 Parallel group : yes ❑ no ✓ E.8.1.6 Cross over : yes ✓ no ❑

E.8.1.7 Other : yes ❑ no ✓

E.8.1.7.1 If yes to other, specify :

E.8.2 • If Controlled specify the comparator :

E.8.2.1 - Other medicinal product(s) yes ❑ no ✓

E.8.2.2 - Placebo yes ✓ no ❑

E.8.2.3 - Other	yes ❑ no ✓
E.8.2.3.1 If yes to other specify :	
E.8.3 Single site in the Member State concerned (see also section G) :	yes ✓ no ❑
E.8.4 Multiple sites in the Member State concerned (see also section G) :	yes ❑ no ✓
E.8.4.1 Number of sites anticipated in the Member State concerned :	
E.8.5 Multiple Member States :	yes ❑ no ✓
E.8.5.1 Number of sites anticipated in the Community :	
E.8.6 Does this trial involve countries outside the EU ?	yes ❑ no ✓
E.8.7 Does this trial have a data monitoring committee ?	yes ❑ no ✓

[22] The descriptions of the trial types provided are those recommended in preference to Phases. See page 5 of Community guideline CPMP/ICH/291/95. The development of a new indication after initial approval of a medicine should be considered as a new development plan.

E.8.8 Definition of the end of trial and justification, in the case where it is not the last visit of the last subject undergoing the trial :[23]

end of the trial after the last visit at time point 5 months

E.8.9 Initial estimate of the duration of the trial [24] **(years, months and days):**

E.8.9.1 - in the MS concerned 0 years 5 months 0 days

E.8.9.2 - in all countries concerned by the trial years months days

[23] If not provided in the protocol

[24] From the 1st inclusion until the last visit of the last subject

F. POPULATION OF TRIAL SUBJECTS

**F.1 Age Span**	
F.1.1 Less than 18 years	yes ❑ no ✓
If yes, specify:	
F.1.1.1 In Utero	yes ❑ no ❑
F.1.1.2 Preterm Newborn Infants (up to gestational age <= 37 weeks)	yes ❑ no ❑
F.1.1.3 Newborn (0-27 days)	yes ❑ no ❑
F.1.1.4 Infant and toddler (28 days - 23 months)	yes ❑ no ❑
F.1.1.5 Children (2-11 years)	yes ❑ no ❑
F.1.1.6 Adolescent (12-17 years)	yes ❑ no ❑
F.1.2 Adult (18-65 years)	yes ✓ no ❑
F.1.3 Elderly (> 65 years)	yes ✓ no ❑
**F.2 Gender**	
F.2.1 Female	yes ✓ no ❑
F.2.3 Male	yes ✓ no ❑

**F.3 Group of trial subjects**	
F.3.1 Healthy volunteers	yes ❑ no ✓
F.3.2 Patients	yes ✓ no ❑
F.3.3 Specific vulnerable populations	yes ❑ no ❑
F.3.3.1 - women of child bearing potential	yes ❑ no ✓
F.3.3.2 - women of childbearing potential using contraception	yes ✓ no ❑
F.3.3.3 - pregnant women	yes ❑ no ✓
F.3.3.4 - nursing women	yes ❑ no ✓
F.3.3.5 - emergency situation	yes ❑ no ✓
F.3.3.6 - subjects incapable of giving consent personally	yes ❑ no ✓
F.3.3.6.1 If yes, specify :	
F.3.3.7 - others :	yes ❑ no ✓

F.3.3.7.1 If yes, specify :

F.4 Planned number of subjects to be included :

F.4.1 - in the Member State :40

F.4.2 For a multinational trial:

F.4.2.1 - in the Community :

F.4.2.2 - in the whole clinical trial :

F.5 Plans for treatment or care after a subject has ended his/her participation in the trial[25]If it is different from the expected normal treatment of that condition, please specify (free text) :

---

[25] If not already provided in the protocol

G. CLINICAL TRIAL SITES/INVESTIGATORS IN THE MEMBER STATE CONCERNED BY THIS REQUEST

**G.1. Coordinating investigator** ***(for multicentre trial)*** **and principal investigator** ***(for single centre trial)***	
G.1.1 and G.1.2 and G.1.3 Name :	Joachim Beige
G.1.4 Qualification (MD……….)	MD
G.1.5 Professional address:	Innere Klinik<br>Klinikum St Georg Leipzig<br>Delitzscher Str. 141<br>Leipzig<br>04105<br>GERMANY

G.2. Principal investigators *(for multicentre trial; where necessary, use additional forms)*

G.2.1 and G.2.2 and G.2.3
Name :

G.2.4 Qualification
(MD……….)

G.2.5 Professional address :

G.3. Central technical facilities to be used in the conduct of the trial. Laboratory or other technical facility, in which the measurement or assessment of the main evaluation criteria are centralised (repeat as needed for multiple organisations)

G.3.1 Organisation Department:	Zentrallabor
Organisation Name:	Klinikum St Georg
G.3.2 Name of contact person :	
G.3.3 Address :	Delitzscher Str. 141<br>Leipzig<br>04105<br>GERMANY
G.3.4 Telephone number :	
G.3.5 Duties subcontracted :	Clinical Chemistry<br>Clinical Haematology

G.3. Central technical facilities to be used in the conduct of the trial. Laboratory or other technical facility, in which the measurement or assessment of the main evaluation criteria are centralised (repeat as needed for multiple organisations)

G.3.1 Organisation Department:	
Organisation Name:	Institut für Klinische Pharmakologie der Charite Universitätsmedizin Berlin Campus Mitte
G.3.2 Name of contact person :	Matthias Huber
G.3.3 Address :	Charite Universitätsmedizin Berlin Campus Mitte<br>Berlin<br>12200<br>GERMANY
G.3.4 Telephone number :	
G.3.5 Duties subcontracted :	Analytical Chemistry

G.4. Organisations to whom the sponsor has transferred trial related duties and functions (repeat as needed for multiple organisations)

G.4.1 Has the sponsor transferred any major or all the sponsor's trial related duties and functions to another organisation or third party ?

yes ❑ no ✓

Repeat as necessary for multiple organisations :

G.4.1.1 Organisation Department:

Organisation Name:	Institut für Biometrie und Medizinische Epidemiologie Charite Universitätsmedizin Berlin
G.4.1.2 Name of contact person :	
G.4.1.3 Address :	Hindenburgdamm 30<br>Berlin<br>12203<br>GERMANY
G.4.1.4 Telephone number :	

Duties/functions subcontracted :

G.4.1.5 All tasks of the sponsor	yes ❑ no ✓
G.4.1.6 Monitoring	yes ❑ no ✓
G.4.1.7 Regulatory	yes ❑ no ✓
G.4.1.8 Investigator Recruitment	yes ❑ no ✓
G.4.1.9 IVRS[26] - treatment randomisation	yes ❑ no ✓
G.4.1.10 Data Management	yes ❑ no ✓
G.4.1.11 E-data capture	yes ❑ no ✓
G.4.1.12 SUSAR reporting	yes ❑ no ✓
G.4.1.13 Quality assurance auditing	yes ❑ no ✓
G.4.1.14 Statistical analysis	yes ✓ no ❑
G.4.1.15 Medical writing	yes ❑ no ✓
G.4.1.16 Other duties subcontracted	yes ❑ no ✓

G.4.1.16.1 If Yes to Other please specify :

[26] Interactive Voice Response System : commonly used for randomisation of treatment and controlling the shipment of stock of product.

H. COMPETENT AUTHORITY / ETHICS COMMITTEE IN THE MEMBER STATE CONCERNED BY THIS REQUEST

H.1 Type of application

If this application is addressed to the Competent Authority, please tick the Ethics Committee box and give information on the Ethics committee concerned. If this application is addressed to the Ethics Committee, please tick the Competent Authority box and give information on the Competent Authority concerned.

H.1.1 Competent Authority ❑

H.1.2 Ethics Committee ✓

Information on Competent Authorities / Ethics Committees

H.2.1 Name :	Ethikkommission der Medizinischen Fakultät der Universität Leipzig
Address :	Härtelstr. 16-18<br>Leipzig<br>04107<br>GERMANY

H.2.2 Date of submission : 2006-10-09

H.3 Authorisation/opinion : ❑ H.3.1 to be requested ❑ H.3.2 pending ✓ H.3.3 given

If given, specify:

H.3.3.1 Date of authorisation / opinion: 2006-11-01

✓ H.3.3.2 authorisation accepted / opinion favourable:

❑ H.3.3.3 not accepted / not favourable.

If not acceptable / not favourable, give :

H.3.3.3.1 - the reasons

H.3.3.3.2 - the eventual anticipated date of resubmission :

I. SIGNATURE OF THE APPLICANT IN THE MEMBER STATE

I.1 I hereby confirm that / confirm on behalf of the sponsor (delete which is not applicable) that

- the above information given on this request is correct

- the trial will be conducted according to the protocol, national regulation and the principles of good clinical practice

- It is reasonable for the proposed clinical trial to be undertaken.

- I will submit reports of suspected unexpected serious adverse reactions and safety reports according to applicable guidance.

- I will submit a summary of the final study report to the competent authority and the ethics committee concerned within a maximum 1 year deadline after the end of the study in all countries.

<u>I.3 APPLICANT of the request for the competent authority(as stated in section C1) :</u>

I.3.1 Date :

I.3.2 Signature :[27]

I.3.3 Print name :

---

[27] On an application to the Competent Authority only, the applicant to the Competent Authority needs to sign.
